# Unveiling the immunological landscape of disseminated tuberculosis: a single-cell transcriptome perspective

**DOI:** 10.3389/fimmu.2025.1527592

**Published:** 2025-02-28

**Authors:** Zhen Gong, Hongxiang Xu, Qiao Zhang, Guirong Wang, Lin Fan, Zilu Wang, Lichao Fan, Chang Liu, Yanhong Yu, Zhou Liu, Qiang Zhou, Huasheng Xiao, Rui Hou, Ying Zhao, Yu Chen, Jianping Xie

**Affiliations:** ^1^ Institute of Modern Biopharmaceuticals, School of Life Sciences, Southwest University, Chongqing, China; ^2^ Department of Clinical Laboratory, The Second Affiliated Hospital of Anhui Medical University, Hefei, Anhui, China; ^3^ Department of Clinical Laboratory, Beijing Chest Hospital, Capital Medical University, Beijing Tuberculosis and Thoracic Tumor Institute, Beijing, China; ^4^ Shanghai Clinical Research Center for Tuberculosis, Shanghai Key Lab of Tuberculosis, Shanghai Pulmonary Hospital, Tongji University School of Medicine, Shanghai, China; ^5^ Shenyang Tenth People’s Hospital, Shenyang Chest Hospital, Shenyang, Liaoning, China; ^6^ Shanghai Biotechnology Corporation, Shanghai, China

**Keywords:** hematogenous disseminated tuberculosis, single-cell sequencing, TCR repertoire, T cell exhaustion, CDR3

## Abstract

**Introduction:**

Hematogenous disseminated tuberculosis (DTB) has an unclear etiology that likely involves multiple factors. Understanding the underlying immunological characteristics of DTB is crucial for elucidating its pathogenesis.

**Methods:**

We conducted single-cell RNA transcriptome and T cell receptor (TCR) sequencing on samples from seven DTB patients. Additionally, we integrated and analyzed data from two published profiles of latent TB infection, three active TB cases, and two healthy controls.

**Results:**

Our analysis revealed a significantly higher proportion of inflammatory immune cells (e.g., monocytes and macrophages) in DTB patients, along with a notably lower abundance of various lymphocytes (including T cells, B cells, and plasma cells), suggesting that lymphopenia is a prominent feature of the disease. T cell pseudotime analysis indicated a decrease in the expression of most hypervariable genes over time, pointing to T cell functional exhaustion. Furthermore, a marked absence of mucosal-associated invariant T (MAIT) cells was observed in the peripheral blood of DTB patients. In the TCR repertoire, specific polymorphisms (TRAV9-2, TRAV13-1, TRBV20-1, and TRBV5-1) and dominant clones (TRAJ49, TRBJ2-7, and TRBJ2-1) were identified. Analysis of the complementarity determining region 3 (CDR3) showed that the most frequent combination was TRAV1-2/TRAJ33, with the motif “CAAMD” being significantly reduced in DTB patients.

**Discussion:**

These findings suggest that lymphopenia and T cell exhaustion, along with unique TCR signatures, may play critical roles in DTB pathogenesis. The reduced “CAAMD” motif and altered TCR clonotypes provide novel insights into the complex cellular dynamics associated with the disease, potentially offering new avenues for targeted immunological interventions.

## Graphical Abstract

Graphical abstract of this study. This research focuses on DTB, utilizing single-cell transcriptomics to characterize the immune landscape of peripheral blood mononuclear cells in DTB. Key findings include: substantial enrichment of innate immune cells and reduction in T-cell abundance in DTB patients’ peripheral blood; significant T-cell exhaustion accompanied by a major loss of MAIT cells; and a reduction in specific TCR gene segments in DTB.

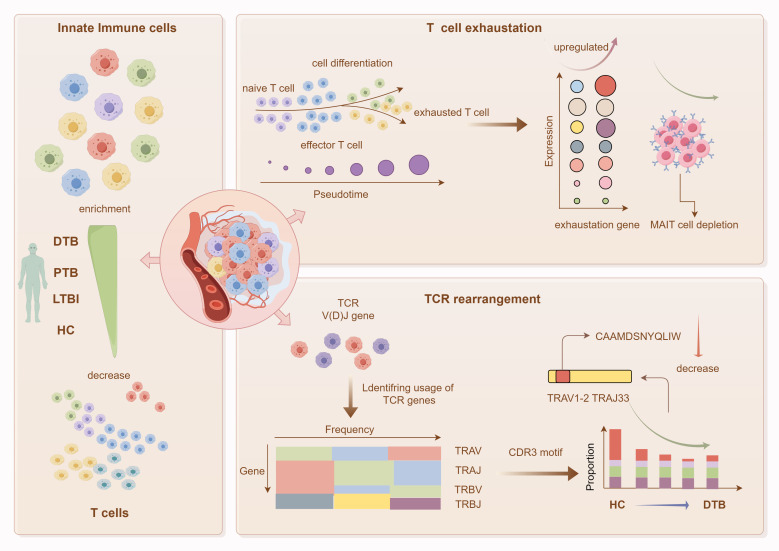

## 1 Introduction

Hematogenous disseminated tuberculosis (DTB), although rare, represents a severe form of tuberculosis in which *Mycobacterium tuberculosis* (Mtb) spreads through the circulatory system to extrapulmonary sites and the central nervous system. Symptoms such as fever, night sweats, fatigue, weight loss, and lymphadenopathy are common, and depending on the affected organs, patients may present with pulmonary symptoms like cough and dyspnea, abdominal issues such as pain and diarrhea, or neurological symptoms including headaches and altered consciousness. DTB typically affects individuals with compromised immune systems, such as those with HIV, organ transplant recipients, or those on immunosuppressive medications. Due to its diverse clinical manifestations, early diagnosis of DTB is challenging, highlighting the importance of understanding its immunological aspects to develop new intervention strategies (1).

A major hurdle in controlling DTB is the absence of accurate biomarkers (2). Single-cell sequencing (Scs) allows for the profiling of gene expression across a wide array of cells, facilitating the identification of novel cell subsets and gene expression patterns (3). Recent applications of Scs in TB research have revealed variations in cell populations in peripheral blood between active TB and latent infections (4), as well as in pulmonary macrophage and monocyte lineages (5, 6). Yet, the characteristics of DTB at the single-cell level remain poorly defined. With the increasing prevalence of older populations, HIV-positive individuals, and patients with diabetes, diagnosing DTB is becoming increasingly complex, underscoring the urgent need for novel diagnostic markers.

In this study, we utilized Scs on peripheral blood mononuclear cells (PBMCs) from seven DTB patients to identify distinct cell types and explore their properties. We conducted pseudotime analysis to examine T cell development, subset heterogeneity, and signs of exhaustion. Our focus also extended to the functional enrichment and expression patterns of differential genes in T cell subsets, alongside investigating TCR development across different DTB patients.

This study uniquely concentrates on DTB, often overshadowed by classical TB, and aims to identify diagnostic markers that hold immense clinical significance, striving to profile peripheral blood cell subpopulations and potential biomarkers at single-cell resolution to enhance timely clinical diagnosis.

## 2 Article types

Original Research

## 3 Manuscript formatting

### 3.1 Methods

#### 3.1.1 Ethical statement

This study was approved by the institutional review board of Shenyang Chest Hospital, China, and informed consent was obtained from each participant.

#### 3.1.2 Subjects/participants and collection of clinical samples

Whole blood samples from patients diagnosed with disseminated tuberculosis (DTB) were collected at Shenyang Chest Hospital. Inclusion criteria encompassed symptoms and signs of tuberculosis, pulmonary lesions indicative of miliary tuberculosis, and imaging evidence of extrapulmonary tuberculosis, including TB meningitis, renal TB, or bone TB. Samples from lesion sites such as sputum, cerebrospinal fluid, urine, bone and joint fluid, and diseased tissue tested positive for *M. tuberculosis* via MGIT960 culture and/or GeneXpert testing. All patients had not undergone previous anti-TB treatment, were HIV negative, lacked confirmed immune deficiency diseases, and were not on immunosuppressive drugs. These criteria ensured the reliability and validity of our study results, as detailed in **Table 1**.

**Table 1:** Clinical and sociodemographic variables for DTB donors.

Number	Gender	Age (years)	Course of disease	Symptom	Diagnosis
DXC0920	male	63	More than 2 years	Cough, expectoration, lumbago, frequent urination, urgency	DTB/Pleurisy/Peritonitis/Urogenital TB/TB of left sacroiliac joint
LGY0920	male	65	More than 1 years	Cough, phlegm, panting, fatigue, swelling and pain of left knee	TB, DTB, TB of left knee joint
GMF1021	male	35	4 months	Emaciated and weak skin changes, and the number of urination increases at night	TB, pleurisy, adrenal TB
WXJ1021	male	53	2 months	Fever, dizziness and headache	TBM, TB, DTB
SMM1021	female	25	More than 50 days	Fever, headache, nausea and vomiting	TBM, TB, DTB,Cervical lymph node TB is more likely
KQL1021	male	58	More than 40 days	Headache and fever	TBM, DTB
CJ1021	male	28	3 months	Fever, fatigue, night sweats, headache	TBM, DTB

The criteria for including patients with miliary tuberculosis (DTB) in this study are:

1. Presence of symptoms and signs of tuberculosis;

2. Pulmonary lesions consistent with radiological changes of DTB, along with imaging manifestations of extrapulmonary tuberculosis in one or more locations, such as tuberculous meningitis, renal tuberculosis, osteal tuberculosis, etc.;

3. Tuberculosis culture (MGIT960) positive and/or detection of MTB by Gene-Xpert in samples (such as sputum, cerebrospinal fluid, urine, synovial fluid, diseased tissue, etc.) taken from the corresponding sites of infection;

4. No anti-tuberculosis treatment prior to inclusion;

5. HIV negative, no immunodeficiency disease, and not using immunosuppressive drugs.

Data from two latent TB infections (LTBI), three active TB cases (PTB), and two healthy controls (HC) were integrated for analysis (Whole blood samples from HC, LTBI, and TB patients were collected at four hospitals in China between August 2018 and October 2019. The initial cohort, comprising 2 healthy controls, 2 LTBI patients, and 3 TB patients, underwent 10x Genomics scRNA-seq). Data sets SRR11038989-SRR11038995 were analyzed (**Supplementary Table S1**). The raw data of DTB can be accessed through the NCBI GEO datasets at the following dataset accession number: GSE287288. Data were harmonized using Harmony, and UMAP dimensionality reduction before and after integration showed effective removal of batch effects while preserving biological differences. Moreover, the biological differences noted by the original authors were still evident after integration.

#### 3.1.3 Single cell sequencing and Data analysis

Whole blood samples were subjected to RNA extraction and single-cell sequencing (Scs) by Shanghai Biotechnology Corporation. Raw data were processed into a cell expression matrix using the Cellranger pipeline (v5.0.0). Data analysis and visualization were conducted using R scripts (v4.2.2) in RStudio. Quality control, dimensionality reduction, and data integration were performed using Seurat (v4) (7). Visualization was carried out using the Uniform Manifold Approximation and Projection (UMAP) method. Samples from different patients and healthy controls were integrated using the IntegrateData method, based on the top 2000 variable genes, scRNA and TCR data for healthy controls were obtained from the 10x Genomics website (dataset: vdj_v1_hs_PBMC_5gex). Cell type identification was conducted using SingleR (8). Pseudotime analysis was conducted using Monocle2, while the Branched Expression Analysis Modeling (BEAM) method identified genes with branch-dependent expression (9, 10). TCR analysis was conducted using the Immunarch package (https://immunarch.com/).

### 3.2 Results

#### 3.2.1 Transcriptional profiles of peripheral blood single cells from patients with DTB

**Figure 1:**
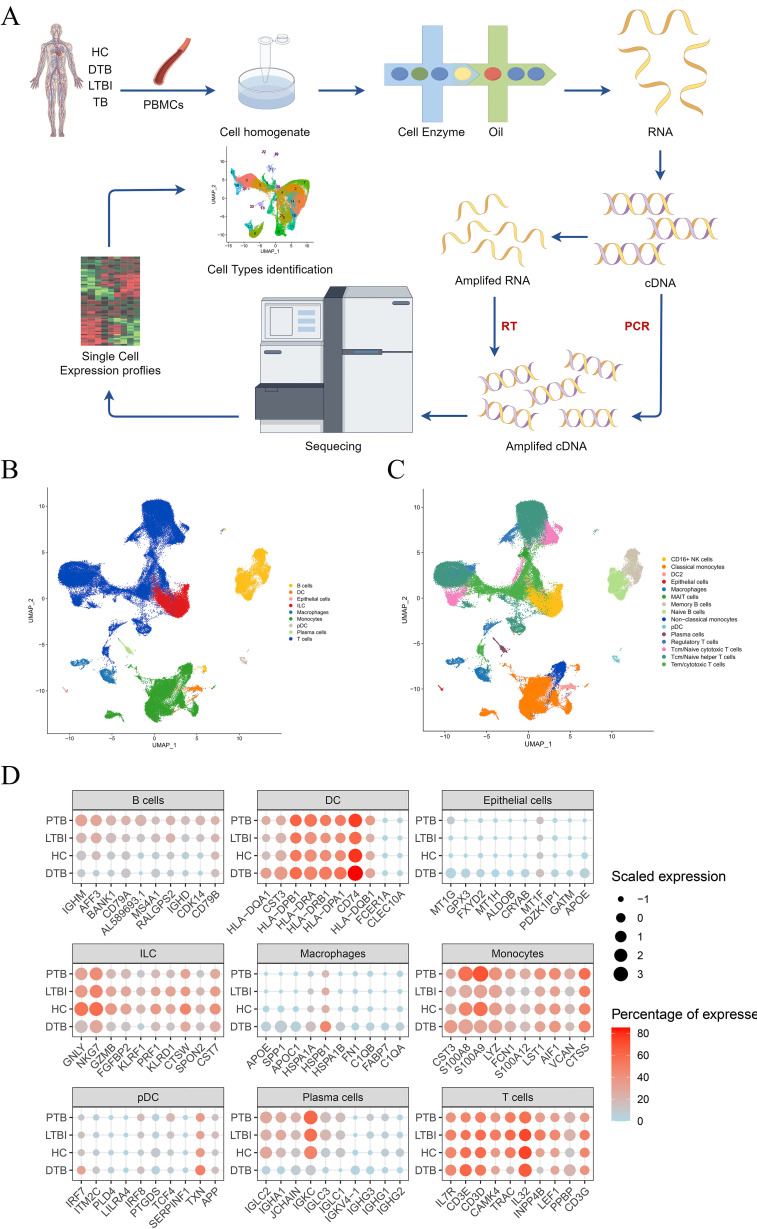
Study Design and Overall Results of Single-Cell Transcriptomic Analysis of PBMCs in Participants. **(A)** Schematic of the overall study design. A total of 7 DTB patients were included, along with integrated data from 2 published studies on latent tuberculosis infection (LTBI), 3 studies on positive tuberculosis (PTB), and 2 healthy controls (HC) for unified analysis. **(B)** UMAP plots depicting the distribution of nine cell types. **(C)** Based on the nine cell subpopulations, cell types were further divided into 15 subgroups. **(D)** Comparison of the expression levels of the top 10 genes with the highest expression in each cell type across DTB, PTB, LTBI, and HC.

To explore the single-cell transcriptional profile during disseminated tuberculosis (DTB) development in detail, we collected peripheral blood samples from seven DTB patients and conducted single-cell RNA sequencing (scRNA-seq) using the 10x Genomics platform, as depicted in **Figure 1A**. By analyzing specific marker gene expressions, we classified these cell clusters into nine major cell types and fifteen cell subsets. UMAP plots clearly demonstrate the distinct distribution of cell types among healthy controls, positive TB, LTBI, and DTB patients. The identified cell types include B cells, T cells, epithelial cells, innate lymphoid cells (ILCs), macrophages, monocytes, plasmacytoid dendritic cells (pDCs), and plasma cells, illustrated in **Figures 1B, C**. Subsequently, we conducted a detailed analysis of cell population proportions and expression levels of the top ten upregulated genes, illustrated in **Figure 1D**. Compared to HC, LTBI, and PTB, there was a significant increase in gene expression of innate immune-related macrophages and monocytes in DTB, whereas the levels of T/B cells and plasma cells decreased.

#### 3.2.2 Gene expression and pseudotime analysis uncover T-cell differentiation traits in DTB

**Figure 2:**
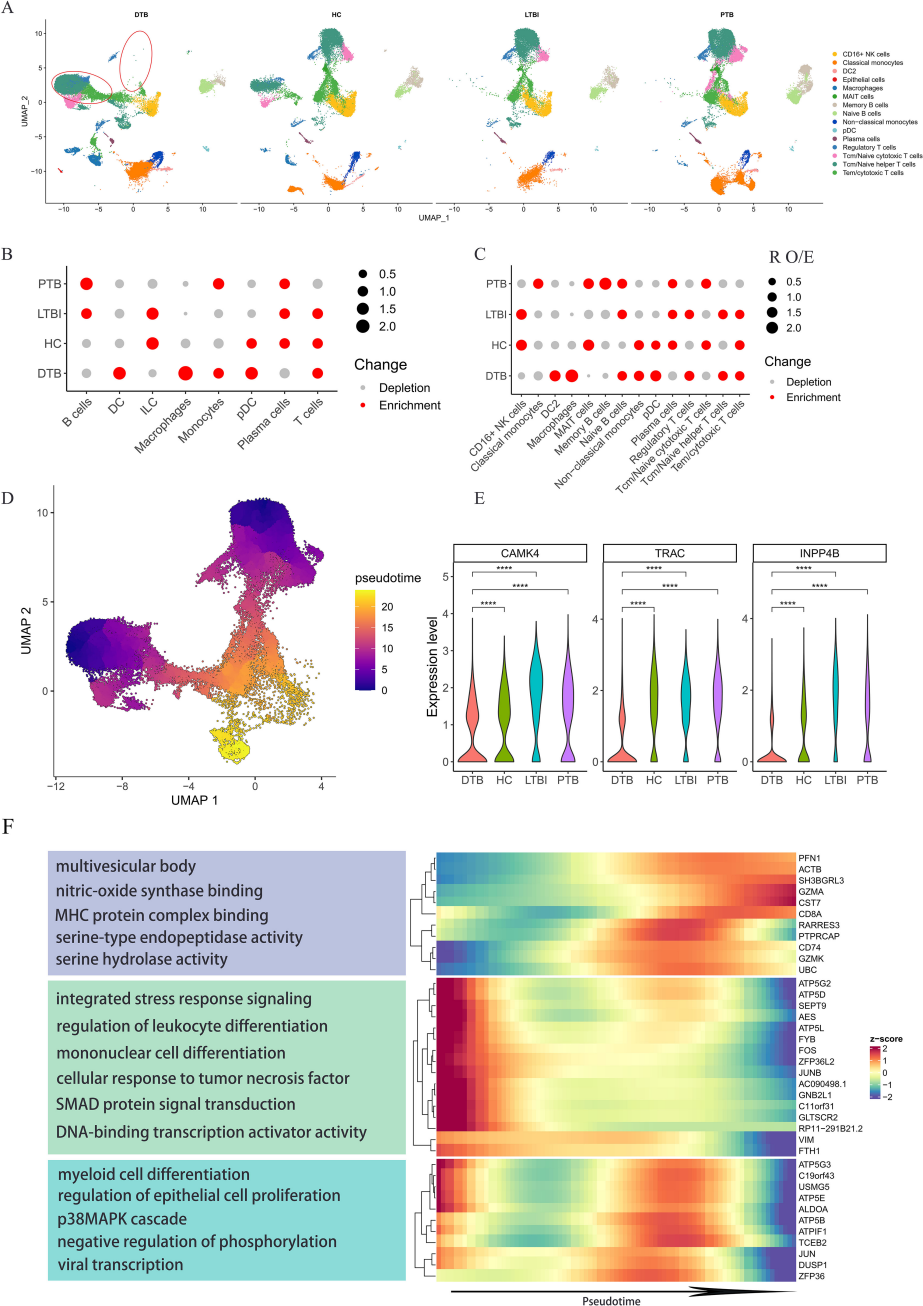
Specific Gene Expression Profiles of T Cell Subpopulations in DTB. **(A)** UMAP mapping of all cells from DTB, PTB, LTBI, and HC shows significant differences in T cell subpopulations in DTB compared to the other three groups. **(B, C)** The preference of each subpopulation for the disease is quantified using the ratio of observed to expected cell counts (Ro/e) across different cell subpopulations. **(D)** Pseudotime trajectory direction for T cell subpopulations is set according to T cell differentiation, progressing from Naive T cells to cytotoxic T cells. The development direction is indicated by colors ranging from dark blue to orange. **(E)** Expression levels of genes related to T cell differentiation among the top 10 expressed genes in T cells across different patients. **(F)** Heatmap and functional enrichment of genes that exhibit high variation and are specifically upregulated in DTB during pseudotime analysis. Expression levels of genes related to T cell differentiation decrease over time. ****: p< 0.0001.

Defining subpopulations across various samples yielded intriguing results. Consistent definitions of cell subgroups across samples indicate stable sequencing results. Surprisingly, the DTB dataset revealed significant disparities in the UMAP representation of T-cell subgroups, indicating unique alterations in T-cell gene expression profiles compared to other samples (**Figure 2A**). We analyzed the expression levels of the top ten genes in T-cells from **Figure 1D**, such as CAMK4, TRAC, INPP4B, and noted a significant reduction in the DTB dataset (**Figure 2E**). We utilized the observed-to-expected cell ratio (R o/e) to quantify each subgroup’s disease association (11). R o/e analysis across all major cell classes and subgroups showed a bias toward DTB in macrophages and monocytes, while cytotoxic T-cells and memory B-cells displayed the opposite trend (**Figures 2B, C**; **Supplementary Figures S1A, B**). The fractional abundance of each subset exhibited certain heterogeneity. Notably, in 2 DTB patient samples, macrophages significantly increased, while changes in the other five samples were less pronounced. Analysis of monocytes and cytotoxic T-cells also revealed a similar trend (**Supplementary Figures S1C, D**). Significant variations were evident in R o/e ratio analysis, whereas changes in cell abundance were less pronounced, possibly due to smaller absolute number changes in these cell subgroups within samples, while their proportional changes were more significant. Additionally, this heterogeneity may reflect the biological complexity of DTB and individual differences in disease progression, immune responses, and other clinical parameters among patients. Given the unique gene expression profile of T-cells in DTB, we conducted a pseudo-time analysis, defining the progression from naive T-cells to cytotoxic T-cells as chronological. The transition of T-cell subgroup colors from dark to light in **Figure 2D** illustrates this chronological order. Ultimately, many genes showed high variability along pseudo-time, with most exhibiting a gradual decrease in expression in DTB as pseudo-time advanced (**Figure 2F**; **Supplementary Figure S2**, and **Supplementary Table S2**). This indicates reduced gene expression with cellular differentiation.

#### 3.2.3 T cell exhaustion in DTB and significant deficiency of MAIT cells

**Figure 3:**
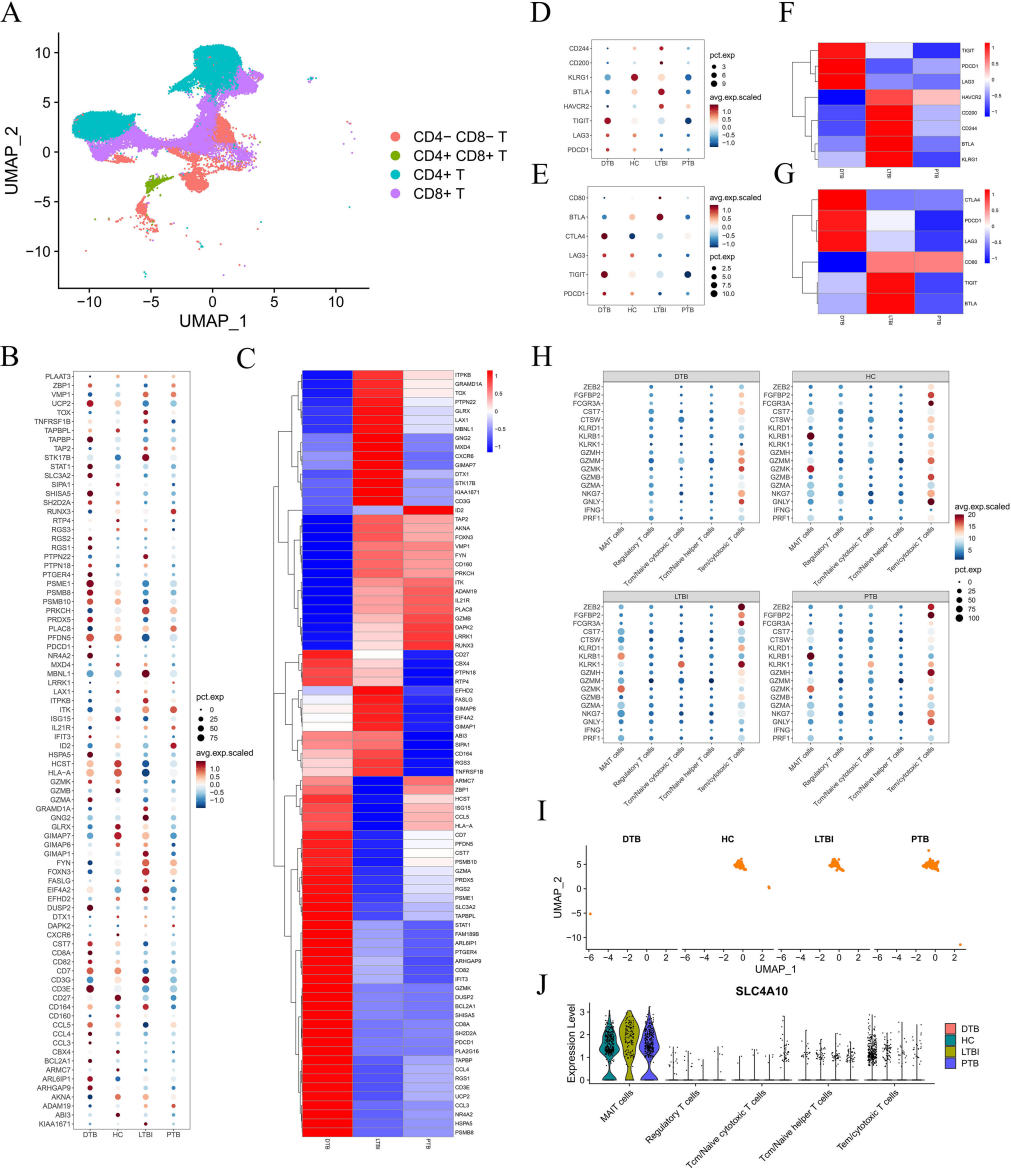
T Cell Exhaustion and Severe Deficiency of MAIT Cells in DTB. **(A)** T cells are divided into four subpopulations based on CD4 and CD8 expression. **(B)** Expression levels of all genes indicative of T cell exhaustion across different patients. **(C)** Heatmap comparing the expression of T cell exhaustion genes in DTB, PTB, and LTBI relative to HC. **(D–G)** Expression levels and comparative heatmaps of genes indicative of CD4+ T cell and CD8+ T cell exhaustion across different patients. **(H)** Dot plot showing the expression of cytotoxicity genes across different patients, indicating a lack of MAIT cell gene expression in DTB. **(I, J)** UMAP mapping of MAIT cells, showing the expression levels of major MAIT cell markers across different patients.

As previously noted, significant changes in T cell gene expression among DTB patients have captured our attention. Most gene expressions gradually decline from naive to cytotoxic T cells, indicating potential T cell functional exhaustion (12). To test this hypothesis, we reclassified T cells as CD4-CD8-, CD4+CD8+, CD4+, and CD8+ using UMAP. **Figure 3A** shows that all samples predominantly contain CD4+ and CD8+ T cells, which exhibit significantly different gene expression profiles in DTB. We then evaluated exhaustion-related gene levels in all patient T cells, using healthy controls as a benchmark, as detailed in **Supplementary Table S3**. The exhaustion gene profile in DTB is generally higher than in PTB and LTBI, consistent with previous findings (**Figures 3B, C**). Specifically, we analyzed these genes in CD4+ and CD8+ cells, observing no significant differences in CD4+ T cells, but elevated levels in CD8+ T cells in DTB (**Figures 3D–G**).

Further analysis yielded surprising results. CD8+ T cells expressed numerous effector molecules targeting *M. tuberculosis*, including PRF1, GNLY, NKG7, GZMA, and GZMB (13). The capacity of CD8+ T cells to release these molecules reflects their functionality. Regulatory T cells (Treg), helper T cells (Th), cytotoxic T lymphocytes (CTL), and other cells express these molecules at varying levels across samples. However, MAIT cells only express certain genes in non-DTB samples, as shown in **Figure 3H**. Given that over 80% of peripheral blood MAIT cells are CD8+, we questioned whether this was due to functional exhaustion or cellular depletion. Subsequent research showed that MAIT cells are nearly absent in DTB samples, with mature surface markers also undetectable (**Figures 3I, J**), indicating a significant loss of MAIT cells and suggesting marked immune dysfunction in DTB patients.

#### 3.2.4 TCR-V(D)J gene rearrangement

**Figure 4:**
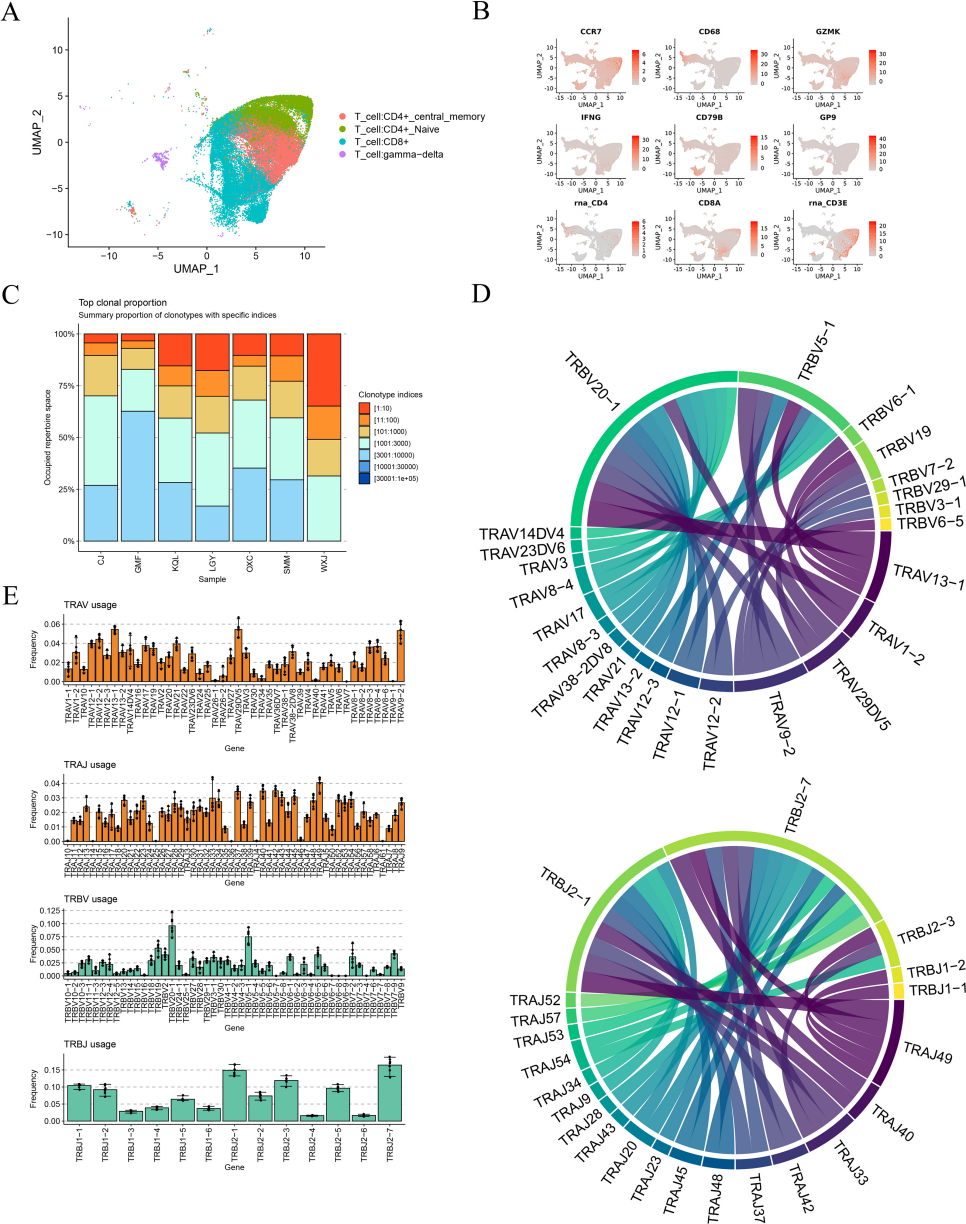
TCR-V(D)J gene rearrangement. **(A)** T cell subtypes. In this study, a total of four subtypes of T cells were obtained: CD4+ naive T cells, CD4+ Central_ Memory T cells, CD8+ T cells, and γδ T cells. **(B)** The expression of T cell function related genes in cell clusters. **(C)** The cloning frequency of TCR in different samples is different, most of which are intermediate frequency sequences. **(D)** The most used TRA-TRBV(D)J genome and type among all samples. **(E)** The most used TRAV(D)J and TRBV(D)J types in all samples.

MAIT cell development relies on TCR rearrangement and interactions with MR1 (14). Numerous studies have indicated that V(D)J rearrangement in T cells occurs in various diseases (15). To explore the clonal relationships between individual T cells and V(D)J gene clones across different samples, we reconstructed the TCR sequence and analyzed the V(D)J genes in DTB patients, as depicted in **Figure 4**.

We conducted UMAP analysis on various T cell subtypes, including CD4+ naive T cells, CD4+ central memory T cells, CD8+ T cells, and γδ T cells, as shown in **Figures 4A, B**. Subsequently, we examined the proportions of TCR clonotypes across different samples, as shown in **Figure 4C**. Intermediate frequency sequences, specifically 1001:3000 and 3001:10000, predominated across all samples. **Figure 4D** displays the top 30 combinations of the most frequently used V and J genes in TRA and TRB.

**Figure 5:**
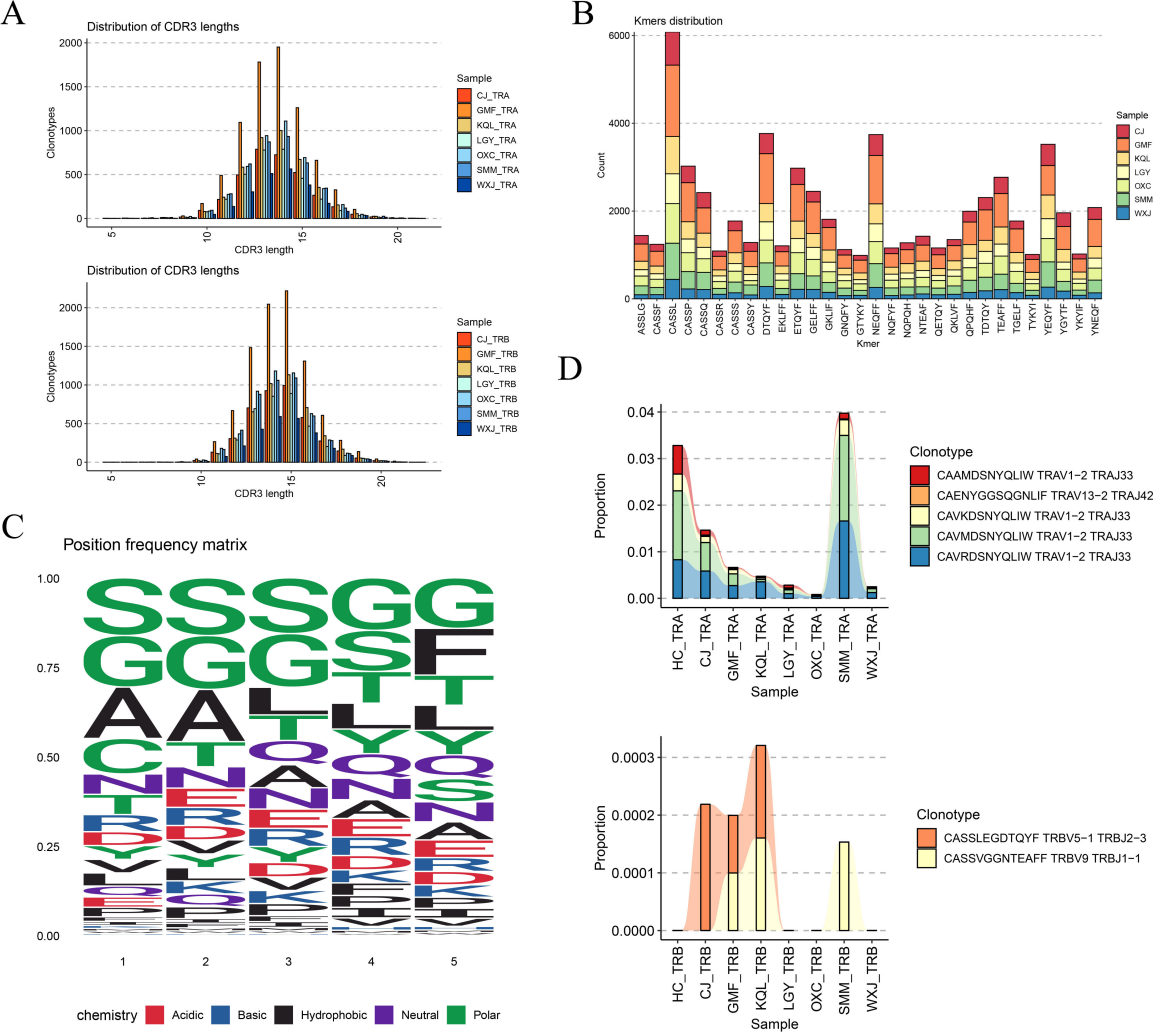
CDR3 sequence usage analysis. **(A)** CDR3 sequence lengths in TRA and TRB were normally distributed. **(B)** The distribution of CDR3 kmers (n =5) in different samples, CASSL has a higher distribution in all samples. **(C)** Frequency of amino acid usage of motif in CDR3. **(D)** Several TCR clonotypes present in all samples.

We analyzed the frequency of V and J gene usage in TRA and TRB. TRAV9-2, TRAV13-1, and TRAV29DV5 were the most commonly utilized V genes in TRA, while TRAJ49 was the most frequently used J gene. In TRB, TRBV20-1 was the most frequently employed V gene, while TRBJ2-7 and TRBJ2-1 were the J genes, as illustrated in **Figure 4E**. These combinations, notably TRBV20-1, TRBJ2-7, and TRBJ2-1, were the most frequently used V and J genes in TRB, aligning with the findings shown in **Figure 4D**. Next, we compared the usage of the complementarity-determining region 3 (CDR3) across all samples. Across the seven samples, most CDR3 sequences were similar in length, ranging from 10 to 15 amino acids. The highest number of CDR3 clonotypes was observed in sample GMF, while sample WXJ displayed the fewest, as indicated in **Figure 5A**, likely due to individual variations.

By segmenting the CDR3 sequence by length (k=5), we obtained Kmer statistical results for the top 30 most frequent CDR3 sequences in TCR. **Figure 5B** illustrates that the proportion of the same fragment across different samples is relatively consistent, with the “CASSL” fragment being the most prevalent. We also analyzed the frequency of amino acid types at different sites within the CDR3 motif, with different colors representing distinct chemical properties of the amino acids, as depicted in **Figure 5C**. Finally, we summarized the common TCR clonotypes observed across different samples. In TRA, TRAV1-2-TRAJ33 and TRAV13-2-TRAJ42 coexisted, with TRAV1-2-TRAJ33 being the most prevalent. In TRB, TRBV5-1 TRBJ2-3 and TRBV9 TRBJ1-1 were present. MAIT cells express a semi-invariant TCR alpha-chain, TRAV1-2-TRAJ33 (16, 17). Compared with healthy controls, the frequency of TRAV1-2-TRAJ33 (CAAMDSNYQLIW) was decreased in all DTB samples, echoing previous findings, as detailed in **Figure 5D**. The relationship between this TCR alpha chain and MAIT function merits further investigation, as illustrated in **Figure 5D**.

### 3.3 Discussion and conclusion

Hematogenous disseminated tuberculosis (DTB) typically manifests with acute onset. This disease frequently involves complications such as tuberculous meningitis, respiratory distress syndrome, and hemophagocytic lymphohistiocytosis syndrome (1, 18), which significantly increase patient mortality. Diagnosing DTB poses significant challenges, and even the most experienced clinicians may encounter difficulties in making accurate judgments. Currently, commonly used diagnostic tools such as ultrasound, CT, and MRI occasionally fail to detect the disease, potentially leading to delays in diagnosis (1). Consequently, there is an urgent clinical need for more precise individual-level diagnostics of DTB.

In this study, we employed scRNA-seq technology to analyze the profiles of peripheral blood cell subpopulations in seven patients with DTB. Additionally, we examined the clonal status of TCRs in DTB patients and explored potential relationships between cell types, TCRs clones, and disease occurrence.

Evidence indicates that early clearance of *M. tuberculosis* infection is linked to a robust innate immune response in resident macrophages. Additionally, recruited monocytes and monocyte-derived macrophages (MDMs) are thought to provide protection during *M. tuberculosis* infection (19). Bone marrow monocytes can differentiate into macrophages that are found in nearly all tissues (20). Recruited blood monocytes can differentiate into macrophages within various tissue microenvironments (21). Macrophages create a suitable niche for *M. tuberculosis*, facilitating its success as a pathogen (22). *M. tuberculosis* exploits macrophage heterogeneity and plasticity to establish and transmit infection. Under drug-induced pressure, *M. tuberculosis* can maintain a latent infection within macrophages (23–25). Monocytes play crucial roles in the innate immune response, with their heterogeneity and ability to differentiate into macrophages or dendritic cells bridging innate and adaptive immune responses (26). An elevated ratio of monocytes and their subsets portends more severe TB symptoms. An elevated ratio of monocytes and their subsets suggests more severe TB symptoms. Comparing DTB with systemic infections such as sepsis, we hypothesize that advanced DTB may cause clinical symptoms and immune profiles similar to those of sepsis, related to the progression of DTB. Monocytes in sepsis show significant heterogeneity, and the immune cell profile changes as sepsis progresses. High immune cell enrichment is observed on day one of sepsis, followed by multicellular exhaustion after one month. Miguel Reyes and others also noted significant differences in the state and abundance of monocytes at different stages of sepsis progression (27–29). Our study observed an increase in non-classical monocytes and a decrease in classical monocytes in DTB patients, consistent with findings by Castano et al (30). Classical monocytes mount an immune response against *M. tuberculosis* during TB infection by enhancing *in vitro* migration to *M. tuberculosis*-derived products, increasing ROS production, lung migration indices, and inducing robust lung infiltration. Immediate infiltration and ROS production by these subsets lead to reduced bacterial growth. In contrast, non-classical monocytes promote bacterial adaptability, exhibit a lower respiratory burst, and lack sufficient CCR2 expression, failing to migrate early to the infection site. Early studies reported an upregulation of CCR2 expression in non-classical monocytes during severe disease to enhance migration to the infection site. Upregulation of CD11b in non-classical monocytes suggests intracellular *M. tuberculosis* survival potential, while loss of HLA-DR leads to inefficient antigen presentation and increased disease severity (31, 32). Disease exacerbation results in changes in the TCR repertoire and affects T cell differentiation, which may have significant clinical implications.

T cells, distributed throughout the body, actively participate in the clearance of foreign substances. CD4+ T cells play a crucial role in maintaining CD8+ T cell responses and in preventing T cell exhaustion (33, 34). The initiation of CD4+ T cell responses to *M. tuberculosis* is notably slow, with CD4+ T cells only reaching the lungs of infected mice several weeks after exposure (35). During host resistance to *M. tuberculosis*, T cells form a complex activation network involving various cell types, including dendritic cells (36), migratory CCR2+ monocytes (37), neutrophils (38–40), and both protective and pathogenic CD4+ T cells. his network also plays roles in Th1 responses (41, 42), the negative regulation of T cell responses, T cell migration, among other functions (43). Theoretically, when *M. tuberculosis* spreads through the bloodstream, free macrophages or phagocytic monocytes rapidly react by phagocytosing the bacteria, presenting antigen epitopes to T cells, and releasing various chemokines to initiate T cell-mediated immune responses. We observed an increase in monocytes and macrophages, while T cells decreased. Cell communication analysis showed active intercellular signaling, playing a crucial role in regulating immune responses and maintaining immune balance (**Supplementary Figure S3**). However, this does not directly prove that the reduction in T cells is directly caused by changes in cell communication. Nonetheless, we hypothesize that changes in cell counts might be related to alterations in cell communication. For instance, due to the pro-inflammatory and anti-inflammatory functions of monocytes and macrophages in chronic inflammatory responses, their increase could indirectly lead to a reduction in T cell counts by secreting specific cytokines that affect T cell survival and function.

In addition to the classical T-helper 1 and T-helper 2 subsets, other subsets such as T-helper 17, regulatory T cells, follicular helper T cells, and T-helper 9 also exist (44). After clearing infectious pathogens, most effector Th cells undergo apoptosis, with the remaining cells contributing to the CD4+ memory T cell pool (45). All memory CD4+ T cell subsets play a crucial role in defense against pathogens (46). CD4+ T cells interact with antigens, which leads to the secretion of cytokines that stimulate CD8+ T cells, thereby facilitating their optimal proliferation and activation (47). CD8+ T cells, functioning effectively, specifically secrete various cytokines to exert immune effects and acquire the ability to lyse cells (48). However, sustained or excessive exposure to antigens can lead to a state of immune exhaustion in T and NK cells, primarily characterized by decreased cytokine secretion, weakened cellular differentiation capacity, alterations in transcriptional profiles, and changes in metabolic pathways (49). In our study, the transcriptional profile of T cells in DTB showed significant alterations, with the expression levels of highly variable genes gradually decreasing over the pseudotime series. Subsequent gene functional enrichment analysis indicated that these genes are primarily involved in various cellular differentiation processes and cytokine signaling pathways, suggesting the potential for T cell exhaustion in DTB.

Comparing the expression trends of highly variable genes in **Figure 2E** with the exhaustion genes in **Figure 3E**, we were surprised to find that most exhaustion-related genes are significantly upregulated in DTB, with their expression levels gradually increasing over the pseudotime series (only a subset of highly variable genes shown), such as CD8A and UBC. A small subset of downregulated exhaustion genes also showed a gradual decrease, such as FYB. It is evident that compared to healthy controls (HC), gene expression levels characterizing T cell exhaustion exhibit systematic variations in DTB, PTB, and LTBI. Genes upregulated in DTB show no significant differences in expression in PTB but are significantly downregulated in LTBI, including ISG15 and CCL5. Conversely, genes downregulated in DTB are both upregulated and downregulated in PTB but are mostly upregulated in LTBI, such as CXCR6 and CD3G, indicating a pattern of change possibly correlated with disease severity. We conducted functional enrichment analysis on these signatures, revealing that genes upregulated in DTB are predominantly associated with chemokine expression activation and receptor interactions, while downregulated genes are involved in T cell differentiation (**Supplementary Tables S4**, **S5**), underscoring the occurrence of T cell functional exhaustion in DTB.

MAIT cells are nearly depleted in the peripheral blood mononuclear cells of DTB patients, a phenomenon confirmed in many severe tuberculosis cases, though not as pronounced as in this study. MAIT cells migrate from the bloodstream to lung tissues and the pleural cavity, suggesting they may move from peripheral blood to local infection sites to exert antimicrobial functions during tuberculosis infections (50). Many tuberculosis infection models demonstrate that MAIT cells rapidly accumulate at infection sites early in the infection, produce inflammatory cytokines, drive the differentiation of dendritic cells derived from monocytes, kill infected cells, and enhance macrophages’ ability to inhibit intracellular MTB proliferation, providing early protective immunity against MTB infection (51, 52). The significant depletion of MAIT cells also indicates that DTB is more severe compared to LTBI and PTB. Combining the expression trends of highly variable genes over pseudotime, the levels of genes related to T cell differentiation gradually decrease over time, leading to an inability to sustain MAIT cell numbers in DTB, and the loss of MAIT cells further impedes the conventional activation pathways of T cell responses. Therefore, the severe depletion of MAIT cells in the peripheral blood of DTB patients may be one of the important immunological characteristics and a significant therapeutic target. MAIT cells, which possess unique markers, have potential clinical value in the diagnosis of DTB. In DTB, the significant reduction of MAIT cells correlates with disease progression, and monitoring these cells can aid in the diagnosis of DTB. Additionally, adoptive immunotherapy, typically used for cancer treatment, can also be applied to treat severe tuberculosis. Within this framework, the strategy of ex vivo expansion and reinfusion of MAIT cells into patients offers a new approach to DTB treatment, utilizing the immune activation capabilities of MAIT cells to combat pathogens, demonstrating a new direction for clinical translation.

The TCR of MAIT cells consists of a conserved Vα7.2 chain, paired with a limited number of TCRβ chains. The depletion of MAIT cells reflects a deficiency in TCR clonotypes. As previously mentioned, the clonal status of TCRs often yields valuable insights into certain diseases. Monoclonal rearrangements of TCR genes are frequently associated with tumors originating from T lymphocyte lineages, such as T-ALL and T/B cell lymphoma (53, 54). Additionally, EBV infection can lead to monoclonal rearrangements of TCR genes, and certain autoimmune diseases may exhibit monoclonal rearrangements of BCR/TCR genes (55). Based on our analysis, the dominant TCR clonotypes observed in the peripheral blood of DTB patients included TRAV9-2, TRAV13-1, TRBV20-1, TRBV5-1, TRAJ49, TRBJ2-7, and TRBJ2-1. Previous studies have indicated a strong association between high expression of TRAV9-2 and Ni2+-mediated allergic contact dermatitis (56, 57), as well as its involvement in celiac disease, a chronic inflammatory disease mediated by T cells (58). Importantly, in active tuberculosis, glycolipid-specific T cells highly express receptors, including TRAV9-2 (59). Notably, TRBV20-1 plays a crucial role in the proliferation of pleural effusion monocytes (PEMCs) and exhibits high expression in TCRs in pleural tuberculosis (60), suggesting that TRBV20-1 may be a prevalent TCR clonotype in TB patients. Other clones have also been reported in various infectious diseases.

The CDR3 region, the most variable region in TCR/BCR, largely determines their specificity. Our analysis across all patients revealed that the motif “CASSL” in TRBV5-1 exhibited a relatively high frequency. Further discussion is necessary to determine if this motif corresponds to a significant tuberculosis antigen. Among all patients, the most frequently utilized TCR was TRAV1-2-TRAJ33. This receptor, expressed by human mucosa-associated invariant T (MAIT) cells, is activated by vitamin B metabolites bound by MR1, a molecule related to the major histocompatibility complex (MHC) class I (61). MAIT cells are abundant in human peripheral blood, comprising approximately 10% of CD3+ cells (62). Previous studies have shown that activation of blood MAIT cells by innate inflammatory cytokines is a primary mechanism in response to *in vitro* stimulation with tuberculosis whole-cell vaccines or mycobacteria (17). Additionally, CD4+ MAIT cells that express IFN-γ and GZMB play a role in anti-TB immunity (63). Our data suggest that CD4+ MAIT cells may be crucial for combating *M. tuberculosis* infection in DTB. Interestingly, TRAV1-2-TRAJ33 is consistently found in both healthy individuals and all patients. However, we observed a significant decrease in the proportion of the specific motif “CAAMDSNYQLIW” within TRAV1-2-TRAJ33 in all DTB patients. It is worth noting that many studies have found that the majority of human MAIT cells express TRAV1-2-TRAJ33. The loss of MAIT cells inevitably affects the levels of TRAV1-2-TRAJ33. Although the specific peptide composition expressed by MAIT cells is not yet clear, our research suggests that “CAAMDSNYQLIW” (TRAV1-2-TRAJ33) may correspond to the deficiency of MAIT cells. Therefore, we speculate that the depletion of the “CAAMDSNYQLIW” (TRAV1-2-TRAJ33) sequence may serve as a potential biomarker for DTB in peripheral blood. In our previous research, we investigated how key sequences of the TCR receptor specifically recognize the Mycobacterium tuberculosis antigen (Mpt). The variability of TRAV1-2-TRAJ33 and its correlation with antigen recognition merit further exploration. Studies like this are expected to advance vaccine development, particularly in the prevention and treatment of tuberculosis.

In conclusion, our study is the first to analyze peripheral blood cell subpopulations in DTB patients at the single-cell level, offering novel insights into the TCR landscape of peripheral blood (**Graphical Abstract**). However, this study also has certain limitations, such as the small number of PTB and LTBI cases included and significant age discrepancies, which could introduce heterogeneity among participants. Due to the rarity of DTB in clinical settings and strict inclusion criteria, our sample size was limited. This limitation might impact the generalizability of our findings. To mitigate this effect, we employed advanced algorithms to minimize heterogeneity across samples. Moreover, by comparing our results with other single-cell tuberculosis data, our findings were further validated, enhancing the significance and relevance of our study. Age differences partially influence the immunological characteristics of human peripheral blood; notably, the loss of naïve CD8+ T cells in elderly individuals (aged 55-65) is a hallmark, yet this is unrelated to innate immune-mediated systemic inflammatory responses. It is noteworthy that MAIT cells are almost nonexistent in the early years of life (<0.08%), increase significantly to about 2.3% in the 5-9 age group, peak at nearly 4.3% among adults aged 19-30, and decrease in individuals over 60 years old (~0.9%) (64). Despite strict inclusion criteria involving older individuals in this study, there were only two participants over 60, and neither was older than 65 years. We believe this age disparity does not significantly impact the overall MAIT cell levels in DTB. Additionally, there are also two individuals over 50 in the LTBI group, which seems not to affect the MAIT levels in LTBI either. Overall, the age heterogeneity in this study does not mislead the research findings. Our main findings are well-supported by other studies, and age disparities seem not to impact these outcomes significantly, it is undeniable that these limitations may have adverse effects (65). Ultimately, This research provides valuable resources for a deeper understanding of peripheral cell subsets in DTB patients and lays the foundation for the rational design of new therapeutic strategies and the discovery of specific vaccines.

## Data Availability

The datasets presented in this study can be found in online repositories. The names of the repository/repositories and accession number(s) can be found in the article/**Supplementary Material**.

## Supplementary material

Supplementary Figure 1 Fractional abundance of subset. **(A)**. Heatmap of Upregulated Genes in Classical Monocytes in DTB. **(B)**. Heatmap of Upregulated Genes in Non-classical Monocytes in DTB. **(C, D)**. Fractional abundance of subset at different resolutions.

Supplementary Figure 2 T cells DEGs GO enrichment in DTB patients. **(A)**. GO enrichment analysis for genes that are upregulated in T cells from DTB patients. **(B)**. GO enrichment analysis for genes that are downregulated in T cells from DTB patients.

Supplementary Figure 3 Cell communication and Ligand-Receptor analysis. Outer circle represents cell types, inner circle represents ligand-receptor pairs; blue represents ligands, red represents receptors, with signals traveling from ligands to receptors as indicated by arrows.
