# Supplementary figures and images for: Unveiling the immunological landscape of disseminated tuberculosis: a single-cell transcriptome perspective
Source: Front Immunol. 2025 Feb 28;16:1527592. doi: 10.3389/fimmu.2025.1527592 (PMC11906432; doi:10.3389/fimmu.2025.1527592)

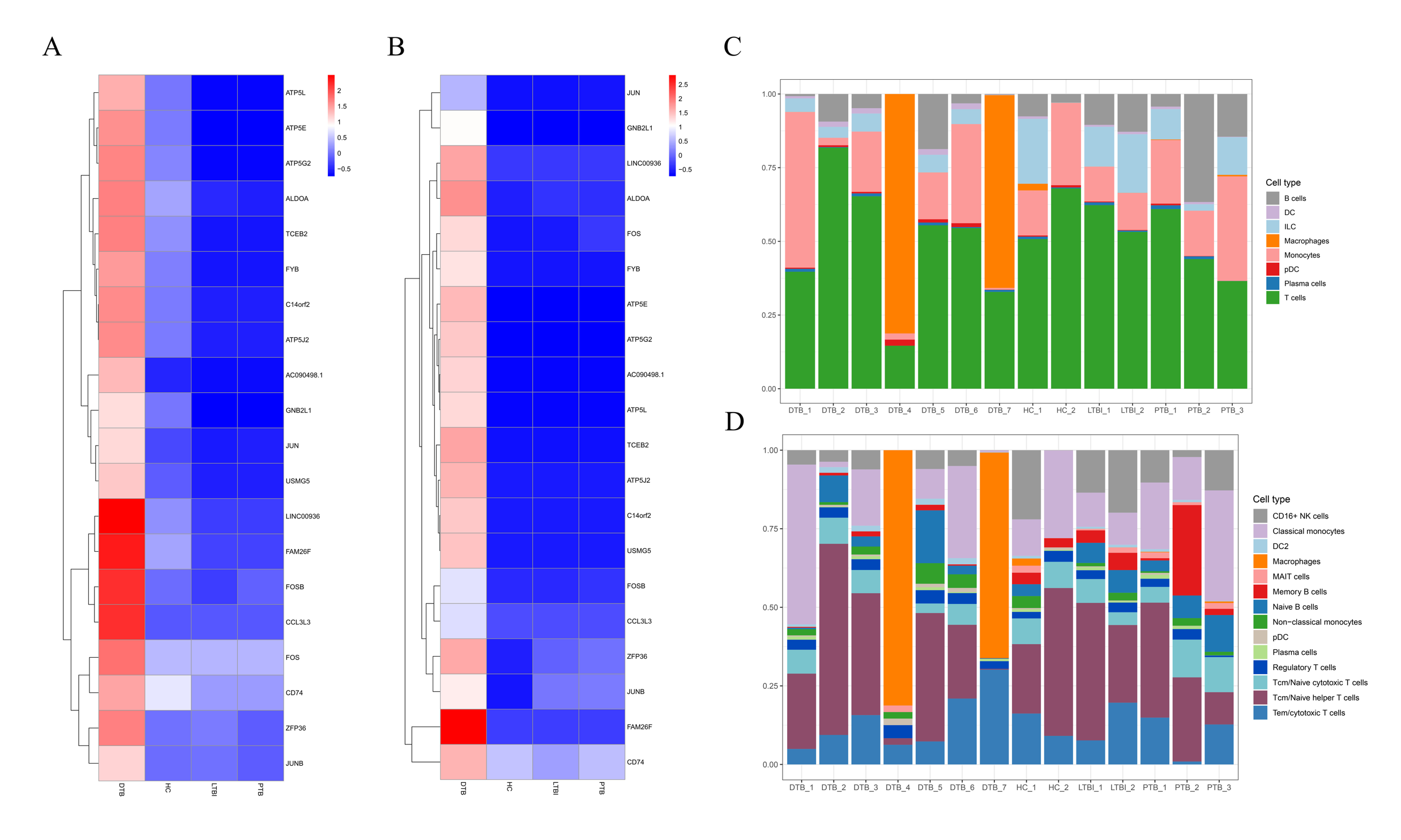

Supplement: Supplementary Figure 1 — Fractional abundance of subset. (A). Heatmap of Upregulated Genes in Classical Monocytes in DTB. (B). Heatmap of Upregulated Genes in Non-classical Monocytes in DTB. (C, D). Fractional abundance of subset at different resolutions. [file Image1.jpeg]

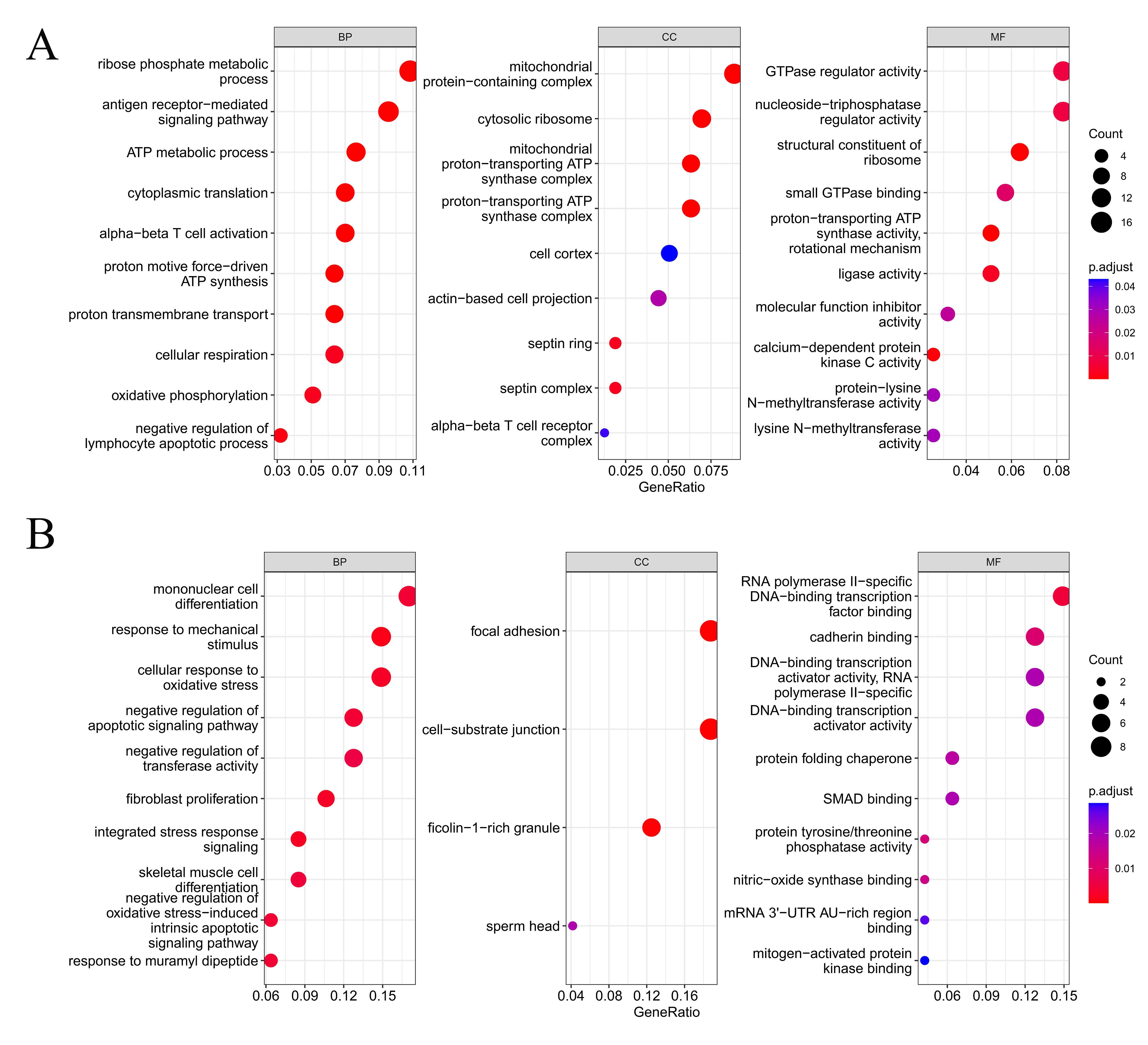

Supplement: Supplementary Figure 2 — T cells DEGs GO enrichment in DTB patients. (A). GO enrichment analysis for genes that are upregulated in T cells from DTB patients. (B). GO enrichment analysis for genes that are downregulated in T cells from DTB patients. [file Image2.jpeg]

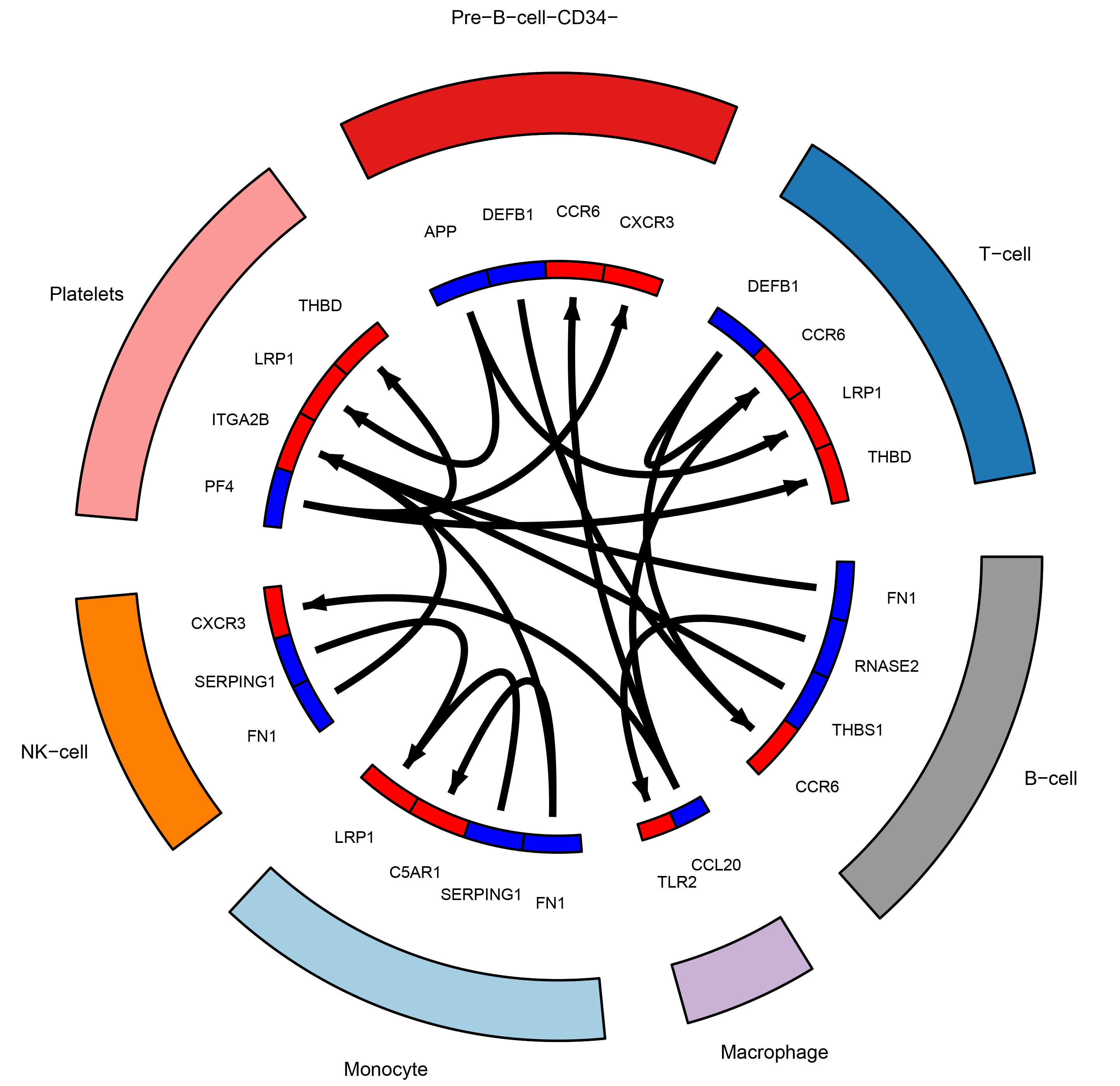

Supplement: Supplementary Figure 3 — Cell communication and Ligand-Receptor analysis. Outer circle represents cell types, inner circle represents ligand-receptor pairs; blue represents ligands, red represents receptors, with signals traveling from ligands to receptors as indicated by arrows. [file Image3.jpeg]
